# Implementing integrated services in routine behavioral health care: primary outcomes from a cluster randomized controlled trial

**DOI:** 10.1186/s12913-019-4624-x

**Published:** 2019-10-24

**Authors:** Mehret T. Assefa, James H. Ford, Eric Osborne, Amy McIlvaine, Ahney King, Kevin Campbell, Booil Jo, Mark P. McGovern

**Affiliations:** 1 Center for Behavioral Health Services and Implementation Research, Division of Public Health & Population Sciences, Department of Psychiatry and Behavioral Sciences, Stanford University School of Medicine, Palo Alto, CA 94304 USA; 2 School of Pharmacy – Social and Administrative Sciences Division, University of Wisconsin – Madison, Madison, WI 53705 USA; 3 Office of Behavioral Health and Managed Care, Division of Behavioral Health and Recovery, Washington State Department of Social and Health Services, Olympia, WA 98504 USA; 4 Washington State Health Care Authority, Olympia, WA 98501 USA; 5 Center for Interdisciplinary Brain Sciences Research, Department of Psychiatry and Behavioral Sciences, Stanford University School of Medicine, Palo Alto, CA 94304 USA

**Keywords:** Implementation research, Co-occurring disorders, Integrated treatment

## Abstract

**Background:**

An estimated 8.2 million adults in the United States live with co-occurring mental health and substance use disorders. Although the benefits of integrated treatment services for persons with co-occurring disorders has been well-established, gaps in access to integrated care persist. Implementation research can address this gap. We evaluated if the Network for the Improvement of Addiction Treatment (NIATx) implementation strategy was effective in increasing integrated services capacity among organizations treating persons with co-occurring disorders.

**Methods:**

This study employed a cluster randomized waitlist control group design. Forty-nine addiction treatment organizations from the State of Washington were randomized into one of two study arms: (1) NIATx strategy (active implementation strategy), or (2) waitlist (control). The primary outcome was a standardized organizational measure of integrated service capability: the Dual Diagnosis in Addiction Treatment (DDCAT) Index. Intent-to-treat analyses and per-protocol analyses were conducted to address the following questions: (1) Is NIATx effective in increasing integrated service capacity? and (2) Are there differences in organizations that actually use NIATx per-protocol versus those that do not?

**Results:**

From baseline to one-year post active implementation, both the NIATx strategy and waitlist arms demonstrated improvements over time in DDCAT Index total and DDCAT dimension scores. In intent-to-treat analyses, a moderate but statistically significant difference in improvement between study arms was seen only in the *Program Milieu* dimension (*p* = 0.020, Cohen’s d = 0.54). In per-protocol analyses, moderate-to-large effects in *Program Milieu* (*p* = 0.002, Cohen’s d = 0.91) and *Continuity of Care* (*p* = 0.026, Cohen’s d = 0.63) dimensions, and in total DDCAT Index (*p* = 0.046, Cohen’s d = 0.51) were found.

**Conclusions:**

Overall, organizations in both study arms improved DDCAT Index scores over time. Organizations in the NIATx strategy arm with full adherence to the NIATx protocol had significantly greater improvements in the primary outcome measure of integrated service capacity for persons with co-occurring disorders.

**Trail registration:**

ClinicalTrials.gov, NCT03007940. Retrospectively registered January 2017

## Background

An estimated 8.2 million adults in the United States live with co-occurring mental health and substance use disorders [1]. The strong association between substance use disorders and other psychiatric disorders is well-documented [2–5]. Research evidence supports the effectiveness of integrated treatment: both substance use and mental health disorders are treated at the same time, during the same treatment episode, and by the same providers [6–11]. The benefits of integrated treatment include, improved health outcomes for patients [12]; higher patient satisfaction levels compared to standard treatment [13]; substantial reduction in utilization and costs of acute care services such as emergency room visits and hospital stays [14]; and cost-effectiveness [15].

Longstanding efforts to improve access to integrated treatment services have been made. However, barriers to delivery of integrated care still persist. The current state of access to adequate treatment for co-occurring disorders remains profoundly limited, and the percentage of specialty addiction programs and mental health programs offering integrated services remain low and highly variable [1, 10, 16–20].

Implementation science may serve to address this gap in treatment access [21]**.** A relatively new discipline, the goal of implementation research is to identify processes and factors related to successful implementation and sustainment of evidence-based practices, programs and policies [22]. Given the lack of treatment availability for co-occurring disorders, a clear need exists to employ implementation research to understand how to scale-up evidence-based integrated treatment effectively [20, 23, 24]**.**

Several studies have demonstrated the effectiveness of the Network for the Improvement of Addiction Treatment (NIATx) for simple practice change in behavioral health settings [25–30]. The NIATx model is a multi-faceted implementation strategy, which combines process improvement with principles from industrial engineering. The process improvement tools and techniques include Plan-Do-Study-Act (PDSA) rapid change cycles and consumer-centered walk-through, and quality improvement interventions include learning sessions, coaching, and interest circle calls [31–33]. However, NIATx has not been evaluated in terms of fidelity or adherence--the extent of key activity completion--or been connected with a range of implementation outcomes. This is the first study to evaluate a well-documented implementation strategy, in this case NIATx, to install and hopefully sustain integrated treatment services for individuals with co-occurring disorders.

The study described aims to address the following research questions: (1) Is NIATx effective in improving integrated services? and (2) Are there differences in organizations that actually use NIATx per-protocol versus those that do not?

Herein, we report primary outcome results from a cluster randomized controlled trial to evaluate the effectiveness of NIATx in implementing integrated services for persons with co-occurring substance use and mental health disorders. The primary outcome measure is the Dual Diagnosis in Addiction Treatment (DDCAT) Index, a widely used instrument to evaluate integrated services capacity at the organizational level. The DDCAT has established psychometric properties, includes an overall total score and subscale scores on seven dimensions that assess policy, clinical practices and workforce domains. It is a comprehensive and objective measure with an established track record of guiding addiction treatment services organizations and systems. In this study, the specific objectives included examination of the primary outcome, DDCAT Index, by conducting: (1) Intent-to-treat analyses by study arm; and (2) Per-protocol analyses by level of NIATx participation. We hypothesized that organizations in the active NIATx study arm would demonstrate greater gains in integrated service capacity, as measured by the DDCAT, compared to the waitlist group.

## Methods

### Design and setting

The study employed a cluster randomized waitlist control group design to evaluate the effectiveness of NIATx in implementing integrated services for persons with co-occurring substance use and mental health disorders. This multi-faceted implementation strategy was used to install and sustain integrated treatment services for programs within community addiction treatment organizations. Agencies were randomized at baseline into either the NIATx strategy or waitlist study arm. NIATx strategies were initiated in the first 12 months for agencies in the NIATx strategy arm, while agencies in the control arm were waitlisted. At the end of year 1, the NIATx strategy group transitioned into the sustainment phase, while the waitlist group began utilizing NIATx strategies. More information on study methods is available in the protocol paper [34].

### Participants

Study participants were programs within community addiction treatment agencies across the State of Washington. Eligibility criteria included: outpatient and/or intensive outpatient services; tax-exempt status; government status or at least 50% publicly funded (e.g., block grants, Medicare, Medicaid); and no prior enrollment in NIATx research studies. In addition, agencies were required to use the state clinical information system to provide the necessary standardized patient-level data. State representatives sent a recruitment letter to all eligible organizations, which included 468 state-licensed addiction treatment providers. In response to this letter, 53 (11.3%) agencies were recruited or volunteered to participate in the study. Four of these agencies declined to continue study participation prior to randomization. The remaining 49 agencies were assigned at baseline to either the NIATx strategy (*n* = 25) or waitlist (*n* = 24) study arms.

### Primary outcome measure

#### DDCAT index

The DDCAT Index (Version 4.0) is a quantitative measure of addiction treatment programs capacity for integrated services for persons with co-occurring substance use and mental health disorders [35]. This organizational measure consists of 35 benchmark items across seven dimensions: (1) *Program Structure*; (2) *Program Milieu*; (3) *Clinical Process: Assessment*; (4) *Clinical Process: Treatment*; (5) *Continuity of Care*; (6) *Staffing*; and (7) *Training*. Each item is rated on a Likert scale ranging from 1 to 5 with scoring anchors of 1 (Addiction Only Services – AOS), 3 (Dual Diagnosis Capable – DDC), and 5 (Dual Diagnosis Enhanced – DDE); an intermediate score of 2 or 4 is given to items that fall between these anchor scores. All items are scored based on data collected by independent evaluators during onsite visits. DDCAT Index dimension and overall scores are derived by calculating the mean of items within a dimension and mean of dimensions, respectively. Using the standard of 80%, addiction treatment programs are categorized as: (1) AOS if less than 80% of scores are rated a 3 or higher; (2) DDC if at least 80% of scores are at a 3 or higher; and (3) DDE if at least 80% of scores are at a 5. Psychometric studies have supported the reliability and validity of the DDCAT Index measure [16, 19, 35–37]. The DDCAT Index Toolkit (Version 4.0) [38] is available at https://www.centerforebp.case.edu/resouces/tools/ddcat-toolkit. The current version of the DDCAT Index measure (Version 4.1) is public domain and available upon request.

To illustrate the characteristics of programs that are categorized as AOS, DDC or DDE, the following brief examples are provided. AOS programs typically either do not screen or treat psychiatric disorders either independent or co-morbid with substance use disorders. The entire focus of the organization’s policy, treatment and workforce is to address substance-related issues only. In fact, AOS programs may exclude patients with known psychiatric disorders from admission. Whereas, DDC programs do provide integrated services for co-occurring psychiatric disorders, but generally only admit patients with a mild to moderate or stable psychiatric condition such as depression or anxiety. Finally, DDE programs typically can provide integrated services to patients with more severe and potentially more acute psychiatric conditions, ranging from depression and anxiety to bipolar and psychotic spectrum diagnoses. DDE programs integrate addiction and mental health services across policy, practice and workforce domains.

### Implementation strategy – NIATx

The NIATx implementation strategy included a coach led site visit, individual coaching calls, group coaching calls and learning sessions (Table 1). For a typical program, the coach made contact approximately two weeks after the DDCAT visit and followed that call up with a site visit planning call two weeks later. Typically, the site visit occurred a month after the site visit planning call but the actual timing was dependent on program staff member availability. The first cohort-wide learning session occurred in October/November which was after the site visit for all but three programs. After the site visit, individual coach calls occurred approximately every 40 days but the actual number of calls varied by program. Two group coaching calls occurred in February and May. The NIATx intervention concluded at the end of June after the wrap-up learning session.

**Table 1:** NIATx Multi-component Implementation Strategy: Components, Timing and Activities

NIATx Strategy Discrete Components	Timing	Activities
Site Visit	Aug to Oct (Cohort 1)	Review the NIATx model
	July to Nov (Cohort 2)	Meet with leadership
		Review the DDCAT scores
		Plan the first change project
Individual Coaching Calls	Approximately monthly from July to June	Review progress of current change projects
		Discuss implementation barriers
		Identify new change projects
Group Coaching Calls	February and May	Discuss common change projects
		Peer to peer sharing
		Discuss sustainability
Learning Sessions	October/November (Initial)	Initial: Apply NIATx strategies to develop PDSA cycles and use data to drive change.
	June (Wrap-up)	Wrap-up: Provider presentations and Sustainment plans

### Procedures

#### Data collection

Data were obtained during independent site visits conducted by evaluators at baseline and one-year follow-up. The evaluators were blind to the study arm. On average, site visits ranged from 3 to 4 h and gathered data via rapid ethnographic observations, key informant interviews and document review. Site visit arrangements were prepared in advance with program leadership. Evaluators conducted brief group and individual interviews with as many program leaders, staff and patients as possible during the half-day visit. Interviews were semi-structured and included participant-specific questions used to elicit information necessary to complete the DDCAT assessment. Document review included extracting information from medical records, brochures, policies and procedures manuals, and other supporting documents. At the end of each site visit, evaluators provided preliminary feedback to program leadership, which was followed up with a formal written report including program strengths, areas for improvement, and DDCAT Index scores. All sources of data were synthesized and summarized to score items on the DDCAT Index. Evaluators independently scored items after each site visit, reviewed together, and discussed to resolve scoring discrepancies.

#### Evaluators

Independent and trained evaluators were from the Washington State Department of Social and Health Services within the Division of Behavioral Health and Recovery (DBHR). A pair of evaluators conducted each site visit independently and one-year post active implementation assessments were completed within a two-month window. All evaluators (*n* = 10) received the same one-day training at the start of the study as well as annual refresher trainings. Trainings incorporated didactic, observational, and experiential approaches, where evaluators observed a site visit and were evaluated conducting a site visit.

#### Ethics

Institutional Review Boards at Stanford University School of Medicine, the University of Wisconsin-Madison, and the State of Washington Department of Social and Health Services reviewed and deemed the study exempt.

### Data analysis

First, we conducted descriptive statistics of baseline characteristics of participating programs.

Next, standard linear mixed effects modeling [39, 40] was employed to estimate changes in DDCAT Index scores from baseline to one-year post active implementation. Following the intent-to-treat principle, all randomized organizations were included in analyses as long as data from at least one of the two assessments/time periods were available. Therefore, a total of 49 organizations in NIATx strategy (*n* = 25) and waitlist (*n* = 24) study arms were included in the longitudinal modeling of the primary outcome, DDCAT Index. Maximum likelihood embedded in the Mplus program Version 8 [41] was used for all model estimations. Specifically, we employed a random intercept model assuming linear change over time.

Initial comparisons included assessment of the estimated trajectories across study arms as randomized (intent-to-treat). In subsequent secondary per-protocol analyses, we compared the two study arms after excluding organizations that were assigned to the NIATx strategy study arm but did not meet the criteria for full participation in NIATx strategies. NIATx participation was determined based on careful consideration of three main components of the Stages of Implementation Completion (SIC), which included: (1) Proportion of completed NIATx activities (e.g., coach calls, webinars, and in-person attendance); (2) Duration of NIATx activities; and (3) Total time from initial to last NIATx activity. Based on these factors, per protocol was defined as organizations in the NIATx strategy group with full adherence (i.e., values above or equal to the average across all three categories) versus organizations in the waitlist group. For full adherence, an agency would complete all NIATx related activities. Since duration of activities and total time are related to the activities completed, full adherence, as measured by duration or total time, is not a construct that can be determined. A univariate GLM examined differences in the three SIC variables based on level of adherence. Given that per-protocol comparisons do not compare groups as randomized, a causal approach known as complier average causal effect (CACE) [42–45] estimation was also employed as a way of sensitivity analysis. In addition, NIATx adherence was examined by the magnitude of DDCAT Index total change scores from baseline to one-year post active implementation. DDCAT Index change categories were defined as: (1) Large positive change (score ≥ 1.5); (2) Moderate positive change (1.5 > score ≥ 0.5); (3) Small positive change (0.0 ≤ score < 0.5); and (4) Negative change (score < 0.0).

## Results

### CONSORT extension for cluster designs

**Fig. 1:**
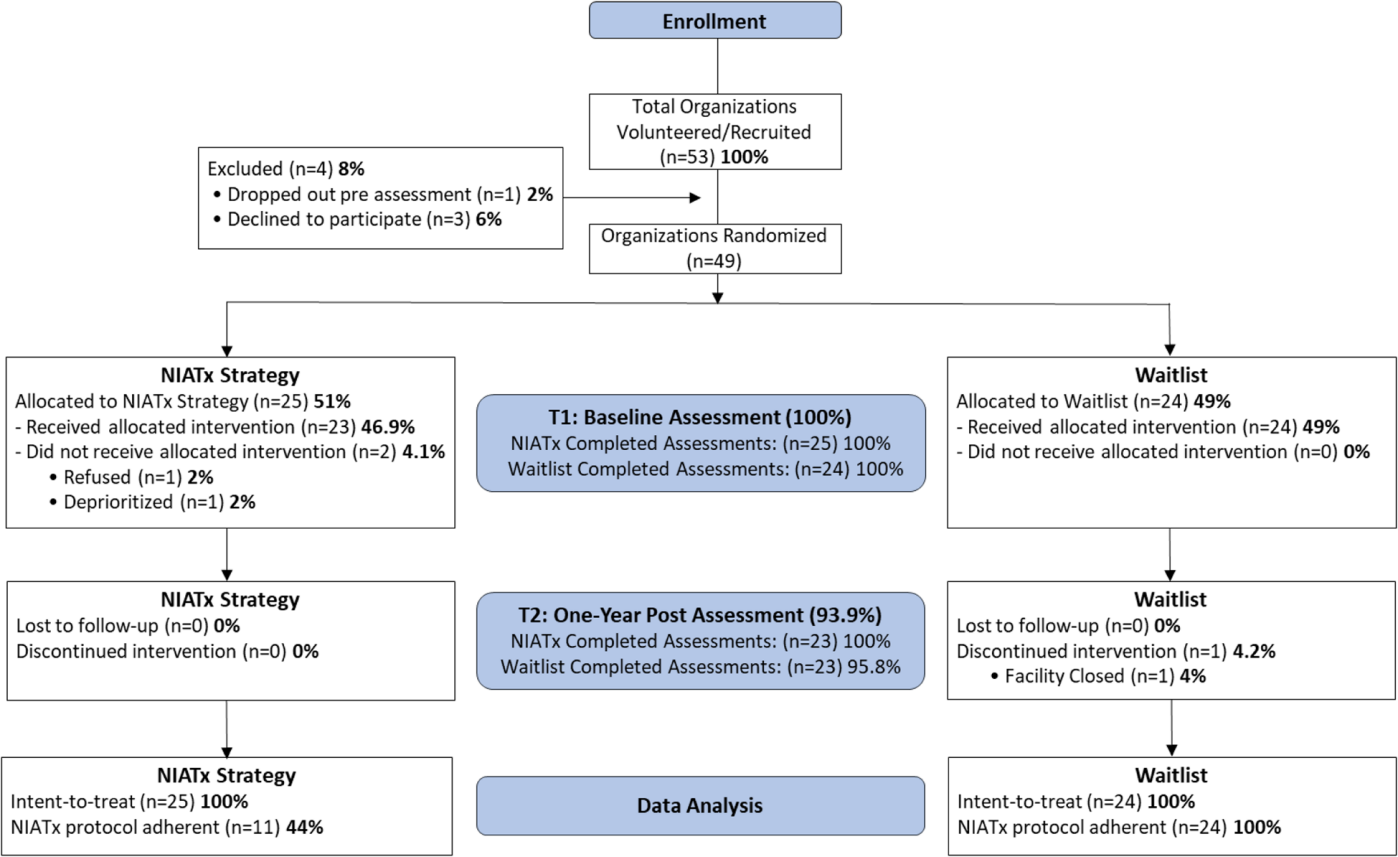
Extended CONSORT Diagram

In 2016, a total of 53 community addiction treatment organizations volunteered or were recruited into the study (Fig. 1). Of those, 49 organizations were randomized to either NIATx strategy (*n* = 25) or waitlist (*n* = 24). At the end of the one-year post active implementation strategy, 23 organizations in each study arm remained. Reasons for dropping out included: deprioritized (*n* = 1), refused ((*n* = 1), and facility closed ((*n* = 1). Follow-up DDCAT assessments were conducted one-year post-baseline, i.e. 2017.

### Baseline characteristics of participating organizations

Overall, the majority of organizations were publicly funded and provided outpatient/intensive outpatient (IOP) care. The addiction treatment agencies were located across the State of Washington in 21 of the 39 counties, located predominantly in cities with medium sized populations (i.e., 26,000 – 249,000). Across the state, of the ten regional behavioral health networks providing funding and treatment services oversite to behavioral health agencies, nine were represented in this study.

Across both study arms, most agencies (55.1%) operated within a medically underserved area. Healthcare shortages in primary care and behavioral health were also identified by participating organizations (71.4 and 75.5%, respectively), with no significant difference by study arm.

### Primary outcome: DDCAT index

Outcomes were analyzed in two ways, with and without consideration of NIATx adherence. Based on the study definition of full NIATx adherence, 13 out of 25 agencies (54%) assigned to NIATx strategy did not show adequate participation (per-protocol) with the intervention.

#### Intent-to-treat comparison of changes in DDCAT index

Intent-to-treat (ITT) analyses were conducted by including all randomized agencies in NIATx strategy (*n* = 25) and waitlist (*n* = 24), regardless of their adherence status. Results from longitudinal mixed effects modeling in line with the ITT principle are summarized in Tables 2 and 3. In Table 3, Cohen’s d is calculated based on observed standard deviation pooled across the NIATx strategy and waitlist study arms at one-year post active implementation. At baseline, organizations in the active NIATx condition arm had higher DDCAT Index total and dimension scores compared to waitlist. Both study arms showed improvements over time in the DDCAT Index total and in all of the seven dimensions scores. However, the two study arms were generally not significantly different in their rate of change, except in the *Program Milieu* dimension, in which organizations in the NIATx strategy arm showed significantly greater improvement in *Program Milieu* compared to the waitlist (*p* = 0.020).

**Table 2:** Estimated DDCAT means at baseline (T1) and post (T2) based on mixed effects modeling

DDCAT Index Dimensions and Total	NIATx Strategy (*n* = 25)	Waitlist (*n* = 24)	NIATx Strategy vs. Waitlist (*n* = 49)
Program Structure T1	3.070	2.489	0.581 (*p* = 0.103)
Program Structure T2	3.830	3.102	0.728 (*p* = 0.051)
Program Milieu T1	2.940	2.562	0.378 (*p* = 0.126)
Program Milieu T2	3.982	3.054	0.928 (*p* = 0.001)
Clinical Process: Assessment T1	3.520	2.970	0.550 (p = 0.005)
Clinical Process: Assessment T2	3.935	3.453	0.482 (*p* = 0.042)
Clinical Process: Treatment T1	2.872	2.342	0.530 (*p* = 0.019)
Clinical Process: Treatment T2	3.612	3.069	0.543 (*p* = 0.016)
Continuity of Care T1	2.872	2.417	0.455 (*p* = 0.087)
Continuity of Care T2	3.640	3.004	0.636 (*p* = 0.013)
Staffing T1	2.936	2.367	0.569 (*p* = 0.060)
Staffing T2	3.689	3.097	0.592 (*p* = 0.021)
Training T1	3.280	2.583	0.697 (*p* = 0.029)
Training T2	4.139	3.467	0.672 (*p* = 0.028)
DDCAT Total T1	3.070	2.533	0.537 (*p* = 0.028)
DDCAT Total T2	3.833	3.177	0.656 (*p* = 0.007)

**Table 3:** Intent-to-treat DDCAT Index change scores by dimension and total (baseline to one-year post) based on mixed effects modeling ((*n* = 49)

DDCAT Index Total and Dimensions	NIATx Strategy ((*n* = 25)	Waitlist ((*n* = 24)	NIATx Strategy vs. Waitlist ((*n* = 49)
Program Structure	0.760***	0.613***	0.147 (d = 0.11)^ƚ^
Program Milieu	1.042***	0.491***	0.551 (d = 0.54)*
Clinical Process: Assessment	0.415**	0.483***	−0.068 (d = 0.08)
Clinical Process: Treatment	0.740***	0.727***	0.012 (d = 0.01)
Continuity of Care	0.768***	0.588***	0.180 (d = 0.20)
Staffing	0.753***	0.730***	0.023 (d = 0.02)
Training	0.859***	0.884***	−0.025 (d = 0.02)
Total	0.763***	0.644***	0.119 (d = 0.14)

*p ≤ 0.05, **p ≤ 0.01, ***p ≤ 0.001

^ƚ^d = Cohen’s d, measure of effect size

**Fig. 2:**
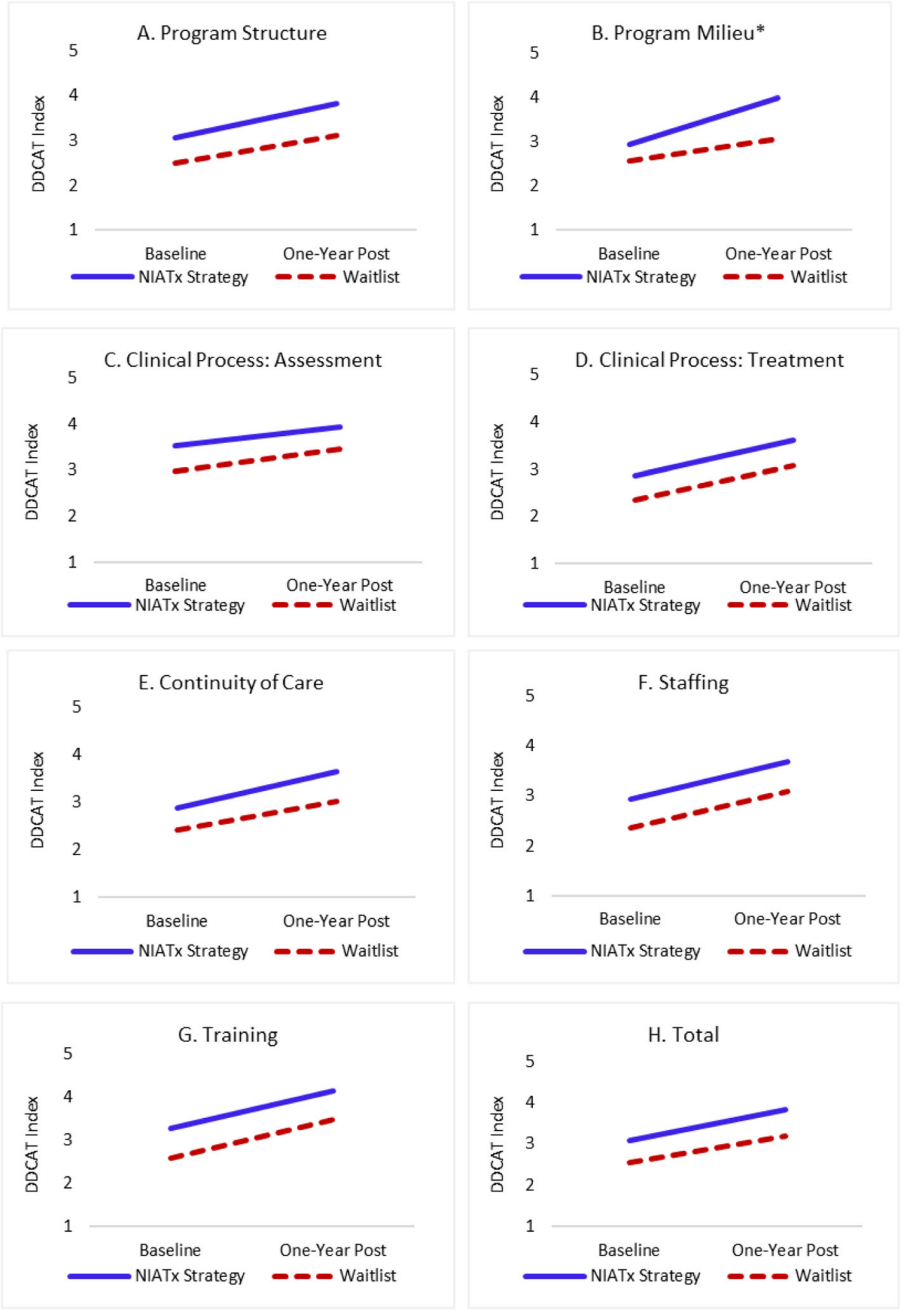
**a**-**h** Intent-to-treat trajectories of DDCAT Index dimension and total change scores (baseline to one-year post) based on mixed effects modeling (*n* = 49) * Group by time interaction effect *p* ≤ 0.05

Figure 2a–h present estimated trajectories of DDCAT Index total and dimension scores based on ITT analyses (graphic depiction of Table 3 data). The two study arms improved similarly in the DDCAT Index total and most dimension scores. In the *Program Milieu* dimension, moderate difference in terms of improvement were found among the two groups. Overall, the effect of NIATx was less evident in ITT comparisons versus the per-protocol analyses presented below. Just less than half (47.8%) of organizations assigned to NIATx were categorized as protocol adherent

#### Per-protocol comparison of changes in DDCAT index accounting for NIATx adherence

**Fig. 3:**
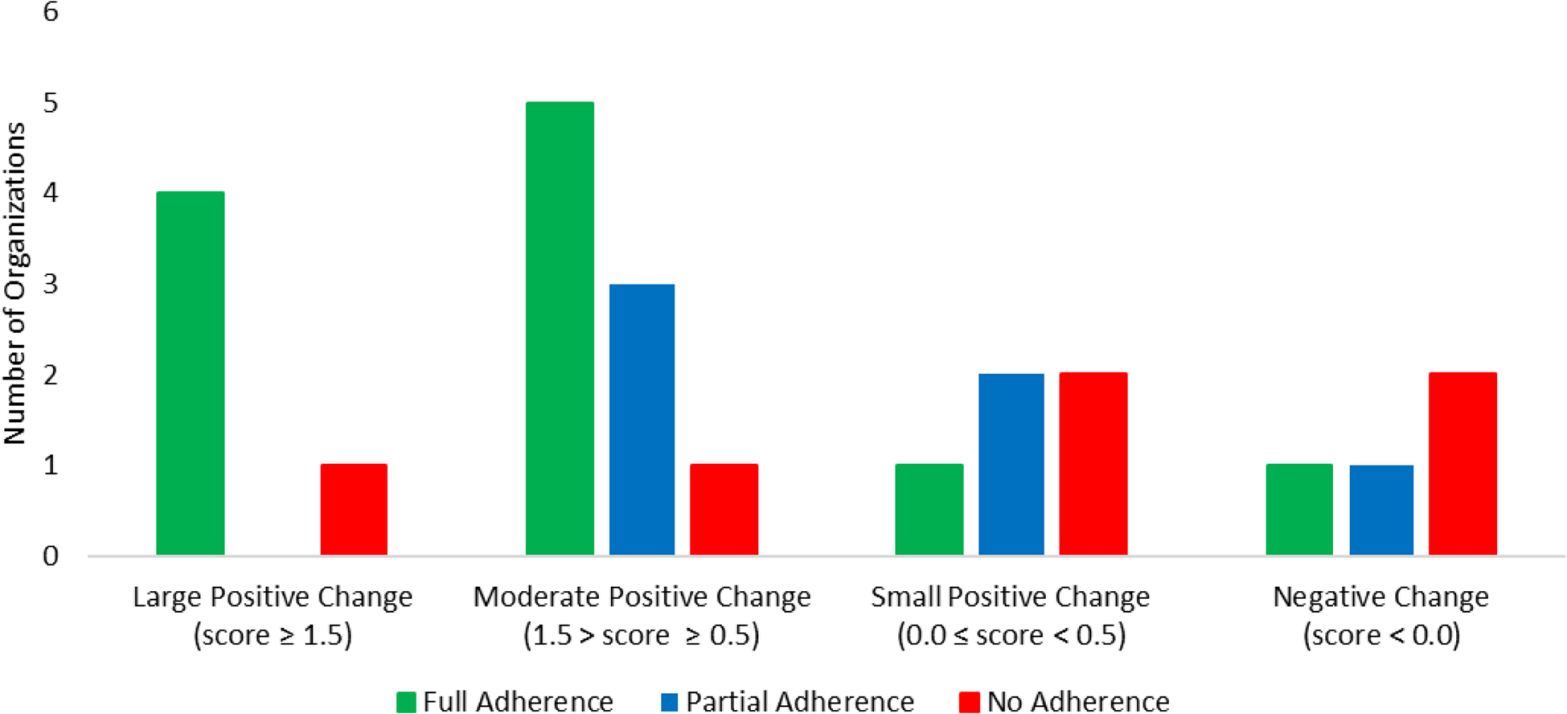
NIATx adherence among organizations by magnitude of DDCAT Index total change score (*n* = 23)

NIATx participation varied between organizations within the NIATx strategy study arm (see Table 4). Of the 23 organizations that completed NIATx strategy, a total of 11 (47.8%) had full NIATx adherence. The remaining organizations had partial or no adherence to NIATx, both 26.1% respectively. Fully NIATx adherent agencies were more likely to complete NIATx activities (85%) versus agencies with no NIATx adherence (41%)(Table 5). Similar significant differences were found for the duration of activities completed (296 versus 128 days) and the total time (291 versus 125 days) between fully adherent and non-adherent NIATx agencies. Partially adherent agencies had a higher proportion of completed activities and a longer duration than non-adherent agencies. Figure 3 depicts NIATx strategy adherence among organizations by magnitude of DDCAT Index total change score. Organizations with large positive (i.e., score ≥ 1.5) and moderate positive (i.e., 1.5 > score ≥ 0.5) DDCAT Index change scores had more organizations with full adherence than those with small positive (i.e., 0.0 ≤ score < 0.5) or negative (i.e., score < 0.0) change scores. Although two of the six organizations with no NIATx adherence had moderate to large change scores, the majority of non-adherent organizations had either small or negative changes.

**Table 4:** NIATx protocol adherence among organizations (*n* = 24)

NIATx Adherence ^ƚ^	Full adherence to NIATx	Partial adherence to NIATx	No adherence to NIATx
NIATx Strategy n (%)	11 (45.8)	7 (29.2)	6 (25.0)
Proportion of Completed Activities ^A^	85.1%	79.5%	41.4%
Duration of Activities (Days) ^B^	295.7	211.1	128.3
Total Time (Days) ^C^	290.6	229.0	124.8
Prior NIATx Experience n (%)	3 (27%)	1 (14%)	1 (17%)
Change Projects Implemented mean (sd)^D^	2.55 (0.93)	1.71 (1.25)	0.83 (0.41)
Number of Coaching Calls mean (sd)^E^	7.00 (2.32)	7.14 (2.97)	2.66 (2.16)
Meeting Attendance ((*n* = 4) n (%)	3.0 (75%)	2.6 (65%)	1.8 (45%)

^ƚ^NIATx adherence consists of three NIATx Stages of Implementation Completion components: 1) proportion of completed activities; 2) duration of activities; and 3) total time from first to last activity. Full adherence to NIATx was any agency with values ≥ the average across all three components; Partial adherence was any agency with values ≤ the average across any two of the three components; and No adherence was any agency with a value ≤ the average across all three components

^A^Significant difference between groups (*F* = 19.77, *p* < 0.001). Full adherence differs from no adherence (*p* = 0.003) and partial adherence differs from no adherence (*p* = 0.007)

^B^Significant difference between groups (*F* = 33.47, *p* < 0.001). Full adherence differs from partial and no adherence and partial adherence differs from no adherence. All *p*-values < 0.001

^C^Significant difference between groups (*F* = 25.43, *p* < 0.001). Full adherence differs from partial (*p* < 0.001) and no adherence (*p* = 0.012). No significant difference between partial and no adherence (*p* = 0.071)

^D^Significant difference between groups (*F* = 5.82, *p* = 0.007). Full adherence differs from no adherence (*p* = 0.005)

^E^Significant difference between groups (*F* = 6.99, *p* = 0.005). Full adherence differs from no adherence (*p* = 0.007) and partial adherence differs from no adherence (*p* = 0.011)

**Table 5:** Per-protocol DDCAT Index change scores by dimension and total (baseline to one-year post) based on mixed effects modeling (*n* = 35)

DDCAT Index Dimensions and Total	NIATx Strategy (Full Adherence) ((*n* = 11)	Waitlist ((*n* = 24)	NIATx Strategy (Full Adherence) vs. Waitlist^ƚ^ ((*n* = 35)
Program Structure	1.136***	0.612***	0.524 (d = 0.39)
Program Milieu	1.409***	0.489***	0.920 (d = 0.91)**
Clinical Process: Assessment	0.740***	0.483***	0.258 (d = 0.31)
Clinical Process: Treatment	1.064***	0.726***	0.338 (d = 0.42)
Continuity of Care	1.164***	0.586***	0.578 (d = 0.63)*
Staffing	0.927***	0.730***	0.197 (d = 0.21)
Training	1.182***	0.885***	0.297 (d = 0.27)
Total	1.089***	0.643***	0.446 (d = 0.51)*

*p ≤ 0.05, **p ≤ 0.01, ***p ≤ 0.001

^ƚ^d = Cohen’s d, measure of effect size

Per-protocol analyses where NIATx strategy organizations showing insufficient adherence were excluded from mixed effects analyses are presented in Table 5. In per-protocol analyses, the difference in improvement for DDCAT Index total scores between the two study arms becomes statistically significant (*p* = 0.046), with a clinically meaningful effect size (Cohen’s d = 0.51). Among the seven DDCAT Index dimensions, *Program Milieu* showed the largest effect (*p* = 0.002, Cohen’s d = 0.91) and *Continuity of Care* showed the second largest effect (*p* = 0.026, Cohen’s d = 0.63).

**Fig. 4:**
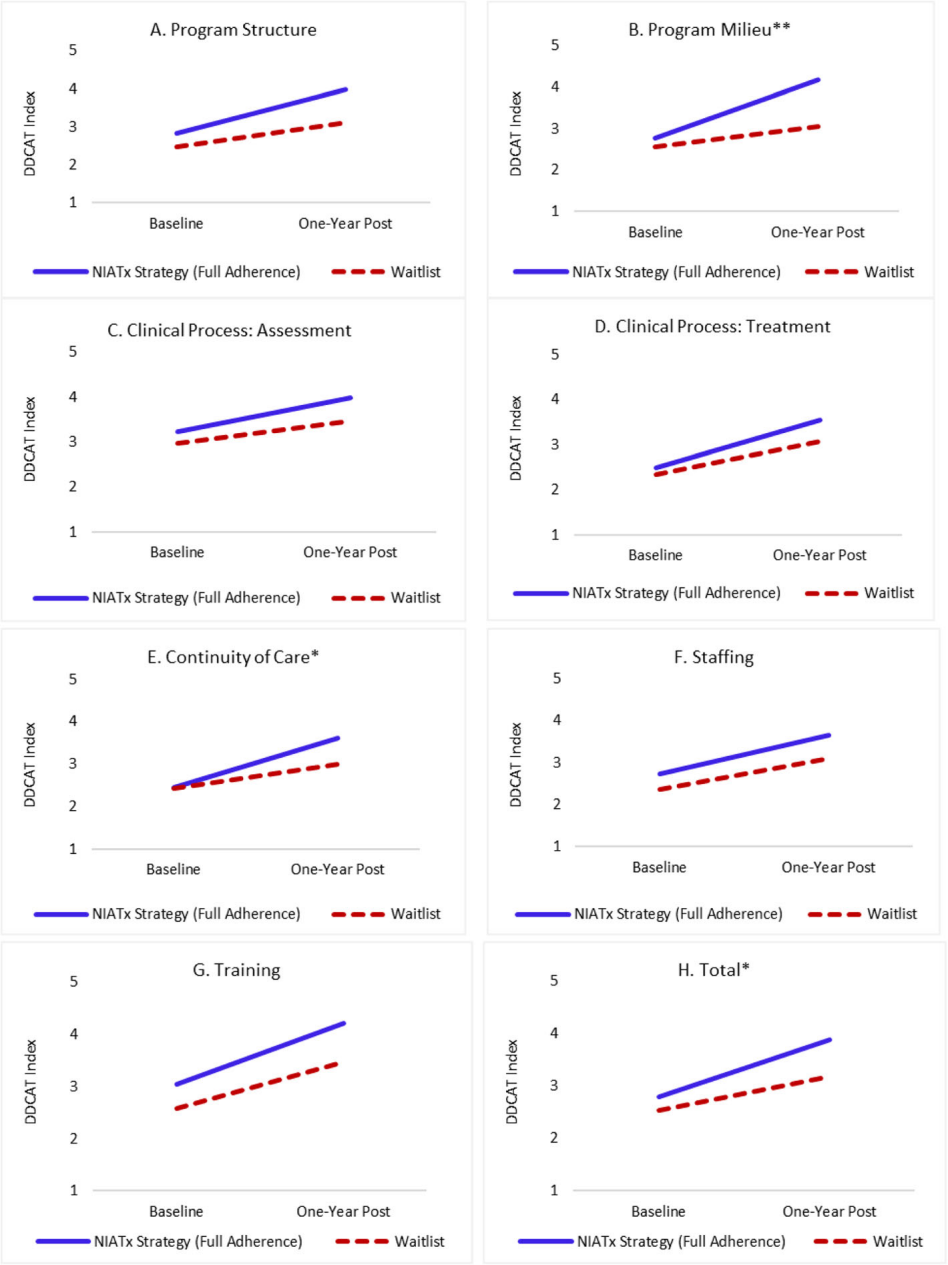
**a**-**h** Per-protocol trajectories of DDCAT Index dimension and total change scores (baseline to one-year post) based on mixed effects modeling (*n* = 49) * Group by time interaction effect (*p* ≤ 0.05); ** Group by time interaction effect (*p* ≤ 0.01)

Figure 4a–h present estimated trajectories of DDCAT Index total and dimension scores based on per-protocol analyses. The comparison between Figs. 2 and 4 illustrates how group differences varied noticeably depending on whether inadequate adherence among organizations in the NIATx strategy group were included or excluded. In per-protocol analyses, the NIATx strategy group improved considerably more than the waitlist group. The two groups show a remarkably large difference in improvement in the *Program Milieu* dimension. Sensitivity analysis using a causal approach known as complier average causal effect (CACE) [42–44] revealed similar results, supporting the validity of the findings based on per-protocol comparisons. There were no statistically significant differences between NIATx adherent and non-adherent programs on baseline characteristics including DDCAT total or dimension scores.

## Discussion

### Summary of findings

Changes in DDCAT Index scores were observed for both study arms. Improvements in DDCAT Index total and dimension scores at one-year post active implementation may indicate that even the audit and feedback of DDCAT Index scores alone, which were also provided to both the active NIATx and the waitlist control group, were useful to initiate important and significant changes in both study arms.

In this study, just over one half of organizations (52.2%) assigned to NIATx strategy failed to sufficiently adhere to NIATx protocol and therefore, fully benefit from the implementation support. Fully adherent agencies reported having prior experience using NIATx implementation strategies (27% vs. 15%) versus non-adherent (i.e. partial or no adherence) agencies. Prior staff knowledge of NIATx benefits may have contributed, on average, to the implementation of more change projects (2.5 vs. 1.3), participation in coaching calls (7.0 vs. 5.1), and greater meeting attendance (75% vs. 55%) for fully adherent agencies versus other non-adherent agencies. In addition, fully adherent agencies were less likely to be located in medically underserved areas or healthcare shortage areas for behavioral health and primary care. Geographic location may have impacted the type and number of change projects implemented by non-adherent organizations. As a result, the differential impact of NIATx was limited via intent-to-treat analyses. Nevertheless, *Program Milieu* showed significant difference with meaningful effect size (d = 0.54). One possible explanation is that within the context of the one-year strategy, some of the implementation focus could have been on *Program Milieu*, such as displaying brochures, and therefore, may have been an easier change to make when compared to more complex integrated treatments or policy and staffing changes found within the other dimensions.

But in so far as adherence to NIATx was taken into account, as demonstrated in the per-protocol analyses results, NIATx implementation strategy effects on DDCAT Index outcomes were more robust (Cohen’s d = 0.51 for DDCAT Index total, d = 0.91 for *Program Milieu* dimension, and d = 0.63 for *Continuity of Care* dimension). Of interest, a few of the other DDCAT Index dimensions, although not statistically significant (perhaps because of other unaccounted variation), still had varying small-to-moderate effect sizes (d = 0.31–0.42).

Previous studies have published results using the DDCAT Index to assess addiction treatment programs across the United States [16, 18, 19, 35–37, 46]. Results have varied, but most report the need for sustained improvements to addiction treatment programs. For example, Lambert-Harris et al. [37], conducted DDCAT Index assessments in 180 community addiction treatment programs. In this study, most programs (81.8%) offered addition-only services. In yet another study [18], approximately 18% of the 256 addiction treatment programs across the United States met the criteria for dual diagnosis capable services. A study of 30 California treatment programs found that 43% of the programs were at dual diagnosis capable or higher, but still faced ongoing barriers to overcome [46]. In this sample, the majority of NIATx strategy and waitlist organizations met the criteria for addiction-only services at baseline (61 and 83%, respectively). However, by the end of one-year follow-up, approximately 22% of NIATx strategy organization provided dual diagnosis enhanced services and only 26% still providing addiction-only services. The waitlist arm also saw a reduction in the number of organizations providing addiction-only services (52%). For both arms, these improvements are meaningful. Two-year follow-up data is needed to determine if organizations in the NIATx strategy are able to sustain improvements and if those in the waitlist are able to make substantial improvements.

### Strengths and limitations

One strength of the study is the experimental design, including randomization at the organizational level. Another strength is the robust primary outcome measure. Furthermore, to date, this is the first study to evaluate a well-documented implementation strategy, NIATx, to install and hopefully sustain integrated treatment services.

The study had some limitations. First, sampling biases due to volunteer or Hawthorne effects were possible. Because of keen interest among participating organizations to integrate behavioral health services in both groups, there may have been some volunteer bias.

Second, because of the convenience sampling used, the external validity of the finds will depend on future studies in different populations. Replication in other settings are needed to verify internal validity.

The DDCAT has excellent psychometric properties and higher scores associated with higher rates of integrated services delivery and improved outcomes for patients with co-occurring disorders. However, in this study it served as the omnibus and proxy outcome of integrated service capacity. Additional measures of outcome would add to the strength of interpretation of findings.

Lastly, because assessments were conducted by evaluators from the State of Washington, it may have prevented some organizations to speak freely about their progress in the study and reveal relevant information during the course of the half-day site visit. To mitigate this, all evaluators stated clearly the purpose of the study, and that evaluations were conducted in their capacity as members of the research team and not as official state employees.

## Conclusions

Many of the agencies enrolled in the study were “early adopters” and participation in the study was partially motivated by the announcement of a new state mandate requiring agencies to transition to integrated behavioral health services for persons with co-occurring disorders. This is evident by the overall improvements found in DDCAT Index scores over time. This level of interest among participating organizations in implementing integrated behavioral health services might also explain the positive DDCAT Index change scores, galvanizing not only organizations in the NIATx strategy arm, but also those in the waitlist. Therefore, these study findings may be specific to this setting. Replication in different contexts and settings may be warranted.

An important finding is that there were hardly robust differences between the NIATx and the waitlist groups on change in integrated service capability, as measured by the DDCAT, using intent-to-treat analyses. Baseline differences between the groups were not eliminated by randomization, so it’s possible that ceiling effects may have undermined a fair comparison. However, if examining the impact of NIATx adherence or participation, much like the dose response effect with medications or psychosocial treatments, more differences between the groups were displayed. Future research might consider designs such as Sequential Multiple Assignment Randomized Trial, such as utilized by Kilbourne et al. [47]. Measuring the impact of an initial discrete strategy (e.g. DDCAT assessment as audit and feedback), and then adapting strategies based on primary outcome measure response or participant adherence, would add a level of rigor and real-world application that this project did not feature.

This study is currently completing two-year follow-up data. Further analysis on sustainment of improvements in follow-up assessments can be examined, and the impact of active implementation for organizations in the waitlist group remains to be seen.

To summarize, providing integrated treatment for persons with co-occurring disorders is important. Its benefits have been demonstrated and yet gaps in integrated services persist. Our findings show that NIATx is effective in implementing integrated services for persons with co-occurring substance use and mental health disorders. It also demonstrates the importance of adherence to the NIATx protocol for significant improvements to be made. To evaluate whether these improvements in DDCAT Index scores correlate with improved patient outcomes, additional analyses will be conducted. Possible implications for behavioral health include determining co-occurring capacity at baseline**,** guiding and measuring evidence-based practice implementation initiatives, and improving patient outcomes.

## Data Availability

Datasets generated and/or analyzed during the current study are not publically available, but are available from the corresponding author on reasonable request. The DDCAT Index measure (V4.1) is available from the authors upon request.

## Abbreviations

CACE: Complier average causal effect
DDCAT: Dual diagnosis capability in addiction treatment
DSHS: Department of social and health services
IOP: Intensive outpatient
IRB: Institutional review board
NIATx: Network for the Improvement of addiction treatment
NIDA: National Institute on drug abuse
PDSA: Plan-Do-Study-Act
SIC: Stages of implementation completion
